# P-1524. Predictive Value of Procalcitonin for Blood Culture Results

**DOI:** 10.1093/ofid/ofaf695.1707

**Published:** 2026-01-11

**Authors:** Masara Touza, Bereket K Tewoldemedhin, Briana crilley, Armando Vidal, Harsh Patel, Mohana Chakkera, Hayley Smith, Maria Akiki, Jihad Slim

**Affiliations:** Saint Michael's Medical Center, Bloomfield, New Jersey; Saint Michaels Medical Center, Newark, New Jersey; Saint Michael's Medical Center, Bloomfield, New Jersey; Saint Michael's Medical Center, Bloomfield, New Jersey; Saint Michael's Medical Center, Bloomfield, New Jersey; Saint Michael's Medical Center, Bloomfield, New Jersey; Saint Michael's Medical Center, Bloomfield, New Jersey; University of Connecticut, Hartford, CT; Saint Michael’s Medical Center, Newark, NJ, USA, Newark, NJ

## Abstract

**Background:**

Early diagnosis and rapid initiation of appropriate therapy are the most effective means of reducing high mortalities in bacteremia and sepsis [1,2]. Blood cultures remain the gold standard for the diagnosis of bloodstream infections [1,2,3] . However, significant lag in blood culture turnover time results in delays of antimicrobial therapy [4]. In certain cases, contaminated samples lead to unnecessary exposure to antimicrobials. Our study tries to evaluate for the diagnostic accuracy of procalcitonin which has been accepted as an excellent marker of pulmonary infections to also be an early indicator of true bacteremia and differentiate between contaminations of blood culture results.

**Table 1:**
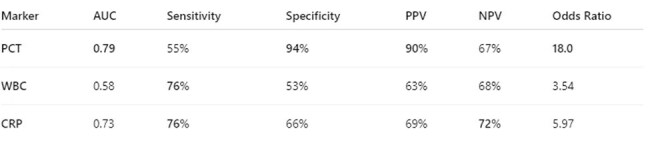
Diagnostic accuracy of procalcitonin, white blood count and C-reactive protein in differentiating true bloodstream infections from contaminated blood cultures.

**Figure 1:**
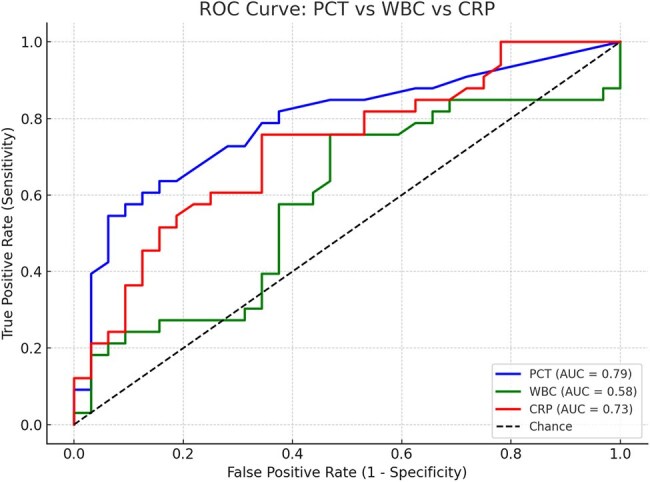
ROC curves comparing procalcitonin, WBC, and C-reactive protein for differentiating true bloodstream infections from contaminated blood cultures.

**Methods:**

A retrospective observational cohort study was done in a large inner city hospital in NJ over an eleven months period. Eligible patients were selected from an existing roster of patients presenting to the ED who are above 18 years old with an anticipated infection requiring blood culture collection prior to initiation of IV antibiotics with concomitant procalcitonin levels drawn at the time of blood culture. Levels were compared with the outcome of the culture data. We excluded factors that could affect procalcitonin levels like CKD (GFR< 35), small cell lung cancer and medullary thyroid cancer, use of IL-2, and antilymphocyte globulins in the past 6 months.

Receiver operating characteristic (ROC) curves were performed to evaluate for the predictive impact of procalcitonin levels as compared to WBC and CRP in patients that have blood cultures positive.

**Results:**

A total of 549 patients met the inclusion criteria and were enrolled. The average age of the patient population was 59. Comparing procalcitonin, WBC and CRP to blood culture results, procalcitonin had the best discriminatory power with high PPV of 90% and specificity 94%, compared to WBC PPV of 63% and CRP PPV of 69%. Our data shows the strongest correlation of procalcitonin above 0.89 ng/ml with blood culture positivity with a p-value of 0.0000387.

**Conclusion:**

Our study adds to the value of using procalcitonin as an early predictor of positive blood cultures. Procalcitonin is the most specific and reliable predictor of blood stream infections compared to other biomarkers, in ruling in bloodstream infections.

**Disclosures:**

Jihad Slim, MD, FACP, gilead: Honoraria|merck: Honoraria|Thera: Honoraria|ViiV: Honoraria

